# Prognostic significance of pretreatment VEGF, survivin, and Smac/DIABLO serum levels in patients with serous ovarian carcinoma

**DOI:** 10.1007/s13277-015-3050-x

**Published:** 2015-01-12

**Authors:** Bozena Dobrzycka, Beata Mackowiak-Matejczyk, Katarzyna Maria Terlikowska, Bozena Kulesza-Bronczyk, Maciej Kinalski, Slawomir Jerzy Terlikowski

**Affiliations:** 1 Department of Obstetrics, Gynecology and Maternity Care, Medical University of Bialystok, 37 Szpitalna Street, 15-295 Bialystok, Poland; 2 Department of Gynecologic Oncology, Bialystok Oncology Center, 12 Ogrodowa Street, 15-027 Bialystok, Poland; 3 Department of Food Science and Technology, Medical University of Bialystok, 37 Szpitalna Street, 15-295 Bialystok, Poland; 4 Department of Gynecology and Obstetrics, Provincial Hospital in Bialystok, 15 Warszawska Street, 15-062 Bialystok, Poland

**Keywords:** Serous ovarian carcinoma, VEGF, Survivin, Smac/DIABLO, Prognostic factors

## Abstract

The second mitochondria-derived activator of caspase (Smac/DIABLO), vascular endothelial growth factor (VEGF), and survivin are known to play a significant role in the growth and development of numerous tumors. Serum concentrations of VEGF, survivin, and Smac/DIABLO were analyzed in 92 patients with serous ovarian cancer and 94 healthy controls. Values were correlated with clinicopathological characteristics and outcomes. The median pretreatment serum VEGF and survivin levels in patients with serous ovarian carcinoma were significantly higher, while Smac/DIABLO levels were significantly lower than that in healthy controls. Receiver operating characteristic (ROC) curve analysis showed that the best cutoff point for VEGF was determined to be 345 pg/ml; with 83 % sensitivity and 65 % specificity. For survivin, the cutoff point was 110 pg/ml and for Smac/DIABLO was 75 pg/ml, with 82 and 62 % sensitivity and 43 and 87 % specificity, respectively. In the patients group, higher VEGF and survivin levels and lower Smac/DIABLO levels in sera were significantly associated with poorer overall survival (OS) and disease-free survival (DFS). Preoperative measurement of serum VEGF, survivin, and Smac/DIABLO may be of help in early detection of serous ovarian cancer and may provide important information about the patient’s outcome and prognosis.

## Introduction

Ovarian cancer is the most lethal cancer among gynecologic malignancies. In 2012, it was estimated that 238,719 cases were diagnosed and 151,905 women died from this disease worldwide [1]. The estimated number of new ovarian cancer cases in Europe in 2012 was 65,538 with 42,704 deaths [2]. In Poland, ovarian cancer is the second most frequent invasive malignancy of the female genital tract after cancers of the uterine corpus, with an estimated 3600 cases diagnosed annually. Approximately 2600 women die each year from ovarian cancer, representing the most common cause of death among women with gynecologic malignancies [3].

Approximately 90 % of malignant ovarian tumors arise from the superficial epithelium which, even though similar to the peritoneal mesothelium, is subject to malignant transformation by genetic modifications that disrupt proliferation and apoptosis [4]. In the group of apoptotic suppressors, inhibitors of apoptosis (IAP) family comprises survivin which is capable of inhibiting the caspases cascade. Cellular apoptosis is directed by two pathways. The extrinsic one is essential for immune selection and inflammation. It commences by the activation of cell death receptors, among them tumor necrosis factor alpha (TNF-α) receptor, which is located at the cell membrane. The intrinsic pathway is set off by toxic abuse such as radiation or chemotherapy (DNA damaging and antimicrotubule agents) [5]. While activating the intrinsic approach, mitochondrial permeability rises, which results in the release of second mitochondria-derived activator of caspase (Smac/DIABLO) (direct inhibitor of apoptosis-binding protein with LOw pI). This proapoptotic protein has a share in both apoptotic pathways, intrinsic and extrinsic. Mature Smac/DIABLO finds its place in mitochondria, and then an apoptotic incentive is released into the cytosol. There, it binds IAPs and neutralizes its inhibitory action on caspases [6]. Survivin is present in many different subcellular locations such as the mitochondria, cytoplasm, and nucleus. However, it has also been spotted in the extracellular space. Extracellular volume can manage to mediate a prosurvival field effect due to the fact that it is secreted by cancer cells and absorbed by surrounding normal and modified cells. By linking extracellular survivin’s capability to boost cellular proliferation, survival, and tumor cell invasion with a membrane-protective trafficking modality, we can definitely provide additional support for the hypothesis that survivin plays a significant role in the cancer cell growth and protection from therapeutic procedures [7]. Survivin is suppressed by Smac/DIABLO which causes the displacement of bound IAPs, which in turn is likely to bind to and subdue caspase function [8].

Tumor growth relies upon its ability to switch the angiogenesis process. Tumoral angiogenesis is the consequence of an imbalance between proangiogenic and antiangiogenic factors. Especially, vascular endothelial growth factor (VEGF) is expressed to a great extent in the majority of ovarian cancers and its risen serum levels as well as tumor VEGF levels have been found to be independent markers of poor clinical outcome [9]. In ovarian cancer, the importance of VEGF serum levels and apoptosis markers is not clear. However, many reports have indicated that an overexpression of these proteins in tumor tissue of patients with ovarian cancer led to poor prognosis [9, 10].

In the present study, VEGF, survivin, and Smac/DIABLO levels were assessed in preoperative sera of patients with serous ovarian carcinoma and the diagnostic as well as prognostic impact content was estimated.

## Material and methods

### Patients

Ninety-two patients with histologically confirmed primary serous ovarian carcinoma International Federation of Gynecology and Obstetrics (FIGO) stages I–IV were enrolled in this study. Patients were treated at the Department of Gynecology of the Provincial Hospital in Bialystok, Poland, between 2005 and 2009, according to the international treatment guidelines for ovarian cancer patients, including primary cytoreductive surgery followed by platinum-containing chemotherapy [11]. All patients gave written informed consent. The protocol was previously approved by the Bioethical Committee of the Medical University of Bialystok.

Patient charts were reviewed to obtain data regarding age, diagnosis, histology, grade, FIGO stage, presence or absence of ascites, residual disease, operative findings, timing of recurrence, and demise. Optimal cytoreduction was defined as <1-cm residual disease after cytoreductive surgery. The pathology for all of the patients with cancer was reviewed by gynecologic pathologist. Five patients with stage IA ovarian cancer did not receive adjuvant chemotherapy; all of the other patients with invasive ovarian cancer were treated with adjuvant chemotherapy. Response to treatment and diagnosis of recurrence was determined according to RECIST criteria or according to CA125 variations (GCIG-criteria) during follow-up [12, 13]. Patients with recurrence during primary therapy or within 6 months after primary therapy were defined as non-responders [14].

Recurrence was proved by clinical examination and imaging. CA125 was determined in most of the patients, but an isolated increase in this tumor marker was not considered as recurrence. At the end of the treatment, patients were regularly evaluated for evidence of a new recurrence by clinical examination, transvaginal and transabdominal sonography, and CA125 values (if elevated preoperative values applied). A CT/MRI examination was performed if the examinations mentioned above revealed pathological findings. Follow-up data was completed until August 2014 at a median follow-up of 44 months (range 1–106). Six patients were lost to follow-up. The status of each patient was recorded as alive without disease, alive with disease, dead of disease, or dead from other causes.

### Laboratory methods

Blood was drawn at the preoperative visit, and serum was stored at −80 °C until examination. The serum from 94 healthy volunteers (ages 21–72 years; median = 54.7 years) served as controls. Quantification of serum protein levels was performed using an enzyme-linked immunosorbent assay with a commercially available Human VEGF Quantikine® enzyme-linked immunosorbent assay (ELISA) Kit and Human Survivin Quantikine® ELISA Kit, R&D Systems, Minneapolis, MN, USA (DVE00 and DSV00, respectively) and Human Total SMAC/Diablo ELISA Kit Intracellular Novatein Biosciences, Woburn, MA, USA (NR-E10804) according to the manufacturer’s protocol using a microplate LabSystems Multiskan Spectrum Microplate Reader (Thermo Scientific, Waltham, MA, USA). Results were calculated from a standard curve generated by a parametric logistic curve fit and expressed in pg/ml of serum. To correct for variation in platelet counts, VEGF per platelet (pg per 10^6^ platelets) was calculated by dividing serum VEGF concentration (pg/ml) by the platelet count (×10^6^/ml).

The minimum detectable dose (MDD) of survivin was 9.96 pg/ml. The assay ranged from 31.2 to 2000 pg/ml as determined by the manufacturer. The MDD of VEGF ranged from 31.2 to 2000 pg/ml. The mean MDD was 9 pg/ml. The MDD of SMAC/Diablo was 5.4 pg/ml. The assay ranged from 56 to 3600 pg/ml. All test runs were duplicated. The patients’ clinical status was not known by the persons running the assays, and the results of these assays were disclosed to the surgeons only after the patients’ disease status was recorded.

### Statistical analyses

Continuous data are expressed as median (range) and were compared using the Wilcoxon rank-sum test. Categorical data were compared by the *χ*2 test or Fisher’s exact test. The Kruskal-Wallis and Mann-Whitney *U* tests were used to evaluate differences between observations. Receiver operating characteristic (ROC) curves were formed in an attempt to determine the accuracy of VEGF, survivin, and smac/DIABLO in detecting ovarian serous carcinoma. ROC curves were used to establish the best cutoff value in this differentiation using the Youden’s index. Sensitivity, specificity, and positive and negative predictive values were calculated using this threshold. Overall survival (OS) was measured as the time from cancer diagnosis to death or date of last follow-up. For the remission cohort analysis, OS was measured from the time of complete remission. Disease-free survival (DFS) was defined as the time from complete remission to treatment failure including relapse, death, or date at last follow-up. Kaplan-Meier analysis was used to construct OS and DFS curves, and curves were compared by the log-rank test. Cox univariate and multivariate proportional hazard models were used to estimate the hazard ratio for each marker and clinicopathological variables. *p* < 0.05 was considered statistically significant. All statistical analyses were calculated using Statistica software version 10.0PL (StatSoft, Inc., StatSoft Polska Sp. z o.o., Poland).

## Results

Patients’ average age was 56 years (within range 32–76 years). Thirty-three patients (35.9 %) developed low-grade (1 or 2) disease, while 59 patients (64.1 %) had high-grade (3) disease. Twenty-one patients (22.8 %) had low-stage (I or II) disease, whereas 71 patients (77.2 %) developed advanced stage (III or IV) disease. Sixty-one patients (66.3 %) underwent optimal cytoreduction in comparison with 31 patients (33.7 %) who underwent suboptimal cytoreductive surgery. Thirty-five patients (38.1 %) did not have any evidence of ascites, while 57 (61.9 %) had ascites present.

**Fig. 1:**
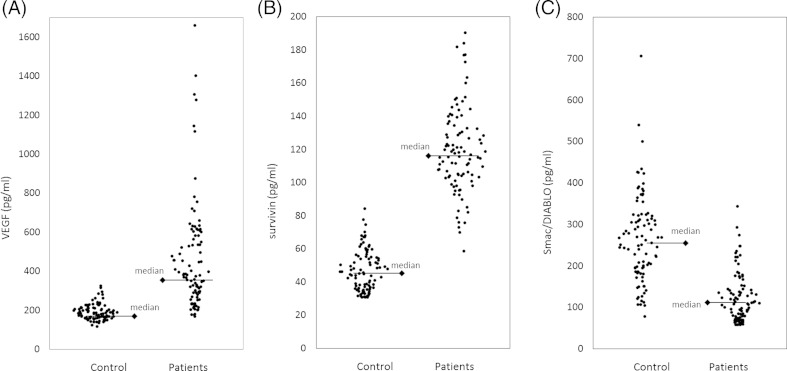
The distribution of serum concentrations of **a** VEGF, **b** survivin, and **c** Smac/DIABLO; individual patient samples

Pretreatment serum VEGF levels ranged from 160.6 to 1611.4 pg/ml, and its median value was 426.8 pg/ml for all the patients who were tested. Serum VEGF levels in healthy controls were also obtained. They ranged from 116.4 to 338.2 pg/ml, with an average value of 186.9 pg/ml. VEGF levels were significantly higher in patients with serous ovarian carcinoma, in comparison with controls (*p* < 0.001). The median platelet count amounted to 224 (in the range of 114–488) × 10^6^/ml. A significant correlation was found between VEGF level and platelet count (*r*_*s*_ = 0.44, *p* = 0.001). Median VEGF level equalled 1.4 (the range between 0.14 and 4.89) pg per 10^6^ platelets. The serum survivin levels in the cancer patient group were found to be in the range from 56.6 to 188.4 pg/ml, with a median value of 112.4 pg/ml, whereas in healthy controls, the range was 32.3 to 86.6 pg/ml and a median value was 44.8 pg/ml, and it showed significant difference (*p* < 0.001). Pretreatment Smac/DIABLO levels were found to be between 56.2 and 328.8 pg/ml, with a median value of 105.6 pg/ml. The level significantly plunged in the serum of patients with cancer in comparison with healthy controls (range from 61.3 to 704.6 pg/ml; median 247.2 pg/ml) (*p* < 0.001). The measurement findings of serum biomarkers in normal healthy controls and serous ovarian carcinoma patients are presented in Table 1. The distribution of serum concentrations of VEGF, survivin, and Smac/DIABLO in individual patient samples is demonstrated in Fig. 1.

**Table 1:** Median concentrations of serum VEGF, survivin, and Smac/DIABLO in serous ovarian carcinoma patients and control group

	Patients (*n* = 92)	Control (*n* = 94)	*p*
	Median (range) (pg/ml)	Median (range) (pg/ml)	
VEGF	426.8 (160.6–1611.4)	186.9 (116.4–338.2)	<0.001
survivin	112.4 (56.6–188.4)	44.8 (32.3–86.6)	<0.001
Smac/DIABLO	105.6 (56.2–328.8)	247.2 (61.3–704.6)	<0.001

We examined the serum levels of VEGF, survivin, and Smac/DIABLO in patients with serous ovarian carcinoma taking into account their age, grade, stage, level of cytoreduction, and presence or absence of ascites. We presented the relationships between these clinicopathological variables and the protein serum levels in Table 2. There was no significant correlation between the levels of these three proteins and patients’ age (*p* > 0.05). VEGF levels were much higher in patients with the advanced tumor stage (*p* = 0.013), grade (*p* = 0.021), ascites (*p* < 0.001), and level of cytoreduction (*p* = 0.009). Serum levels of survivin rose in regard to the advanced tumor stage (*p* = 0.036), grade (*p* = 0.032), ascites (*p* = 0.002), and cytoreduction (*p* = 0.018). The serum levels of Smac/DIABLO fell significantly with the tumor stage and grade (*p* = 0.015 and *p* = 0.034, respectively). We did not find any significant differences in Smac/DIABLO levels based on ascites or ability to achieve optimal level of cytoreduction (*p* = 0.112 and *p* = 0.192, respectively) (Table 2).

**Table 2:** Correlation between the VEGF, survivin, Smac/DIABLO, and clinicopathological parameters

Parameter	VEGF (pg/ml)		survivin (pg/ml)		Smac/DIABLO (pg/ml)	
	Median (range)	*p*	Median (range)	*p*	Median (range)	*p*
Age						
<56	492.6 (160.6–855.2)	0.063	87.2 (56.3–146.8)	0.214	117.8 (68.3–328.6)	0.262
≥56	538.4 (212.4–1611.5)		125.2 (58.4–188.4)		97.8 (57.3–268.4)	
Grade						
1–2	413.8 (160.6–888.5)	0.021	73.2 (56.3–142.4)	0.032	124.3 (64.1–328.6)	0.034
3	559.7 (265.5–1611.5)		131.4 (62.3–188.4)		87.2 (57.3–284.8)	
Stage						
I–II	383.6 (160.6–764.8)	0.013	82.2 (56.3–148.6)	0.036	134.8 (68.8–328.6)	0.015
III–IV	598.4 (448.2–1611.5)		139.5 (64.6–188.4)		74.2 (57.3–276.2)	
Cytoreduction						
≤1 cm	346.2 (160.6–887.4)	0.009	84.2 (56.3–138.8)	0.018	114.4 (58.9–328.6)	0.192
>1 cm	564.2 (269.6–1611.5)		149.4 (66.2–188.4)		94.8 (57.3–245.4)	
Ascites						
Absent	287.8 (160.6–754.8)	<0.001	76.2 (56.3–145.4)	0.002	116.8 (71.3–328.6)	0.112
Present	574.2 (264.5–1611.5)		142.8 (59.2–188.4)		97.6 (57.3–258.2)	

**Fig. 2:**
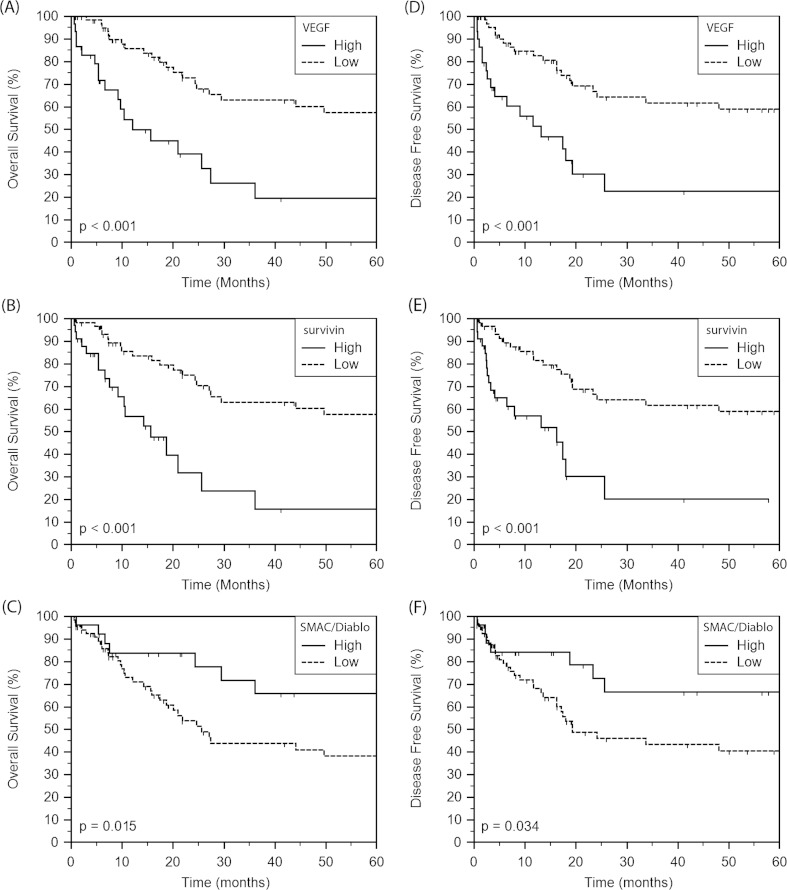
Kaplan-Meier analysis for **a**–**c** OS and **d**–**f** for DFS according to VEGF, survivin, and Smac/DIABLO serum levels. In each set, different subgroups were plotted according to the cutoff value of VEGF, survivin, and Smac/DIABLO levels defined as the median of the set

We analyzed the group of patients for the relationship between the clinical outcome and the levels of VEGF, survivin, and Smac/DIABLO. Kaplan-Meier analyses for OS and DFS were carried out by means of the median levels of proteins as the cutoff for the definition of the subgroups. Higher serum VEGF and survivin or lower Smac/DIABLO levels corresponded with poor prognosis (Fig. 2). The OS probabilities at 5 years of the high VEGF (21 %) and survivin (18 %) group were notably lower than those of the low VEGF (59 %; *p* < 0.001) and survivin (61 %; *p* < 0.001) group (Fig. 2a, b, respectively). The patients in the low Smac/DIABLO group developed significantly shorter OS than those in the high Smac/DIABLO group (*p* = 0.015; Fig. 2c). The probabilities of DFS at 5 years in the low VEGF and survivin group were 62 and 63 %, respectively, while in the high VEGF and survivin group equalled 24 and 22 %, respectively (*p* < 0.001; Fig. 2d, e). There was also an apparent association between low Smac/DIABLO levels and a reduced DFS (*p* = 0.034) (Fig. 2f).

Univariate analysis of the clinicopathological parameters proved that stage, grade, residual tumor, and ascites were found to correlate with a shorter DFS and OS in serous ovarian cancer patients (Table 3).

**Table 3:** Univariate analyses of disease-free and overall survival at 5 years

	Disease-free survival (DFS)			Overall survival (OS)		
	HR	95 % CI	*p*	HR	95 % CI	*p*
VEGF (low vs. high)	2.189	1.284–3.136	<0.001	2.111	1.188–4.943	<0.001
survivin (low vs. high)	2.054	1.182–3.025	<0.001	1.731	1.642–2.843	<0.001
Smac/DIABLO (low vs. high)	1.776	1.044–2.982	0.034	1.442	1.214–2.806	0.015
Age (<56 vs. ≥56)	0.814	0.528–1.082	0.442	0.852	0.584–1.681	0.228
Stage (I/II vs. III/IV)	9.889	2.646–41.431	<0.001	10.618	2.672–42.678	<0.001
Grade (1–2 vs. 3)	1.832	0.732–2.944	0.012	1.824	0.727–2.249	0.034
Cytoreduction (≤1 vs. >1 cm)	2.596	1.382–4.038	0.003	2.289	1.076–4.828	0.006
Ascites (absence vs. presence)	2.687	1.428–4.252	0.011	2.262	1.123–3.712	0.004

In multivariate analysis of the clinicopathological parameters, stage and tumor grade remained to be seen as independent predictors of DFS in ovarian cancer patients. Stage and residual tumor turned out to be independent predictors of OS. Multivariate analysis identified raised serum VEGF as well as survivin and decreased serum Smac/DIABLO levels as independent risk factors for the prognosis (Table 4).

**Table 4:** Multivariate Cox proportional hazards model for disease-free and overall survival at 5 years

	Disease-free survival (DFS)			Overall survival (OS)		
	HR	95 % CI	*p*	HR	95 % CI	*p*
VEGF (low vs. high)	2.648	1.142–3.663	<0.001	2.340	1.186–4.643	<0.001
survivin (low vs. high)	2.073	1.078–3.739	0.001	2.086	1.109–3.942	0.004
Smac/DIABLO (low vs. high)	2.081	1.146–2.866	0.023	2.146	1.208–2.988	0.044
Stage (I/II vs. III/IV)	9.032	2.743–40.352	<0.001	9.936	2.748–41.122	<0.001
Grade (1–2 vs. 3)	1.986	0.746–2.864	0.032	0.842	0.458–1.523	0.528
Cytoreduction (≤1 vs. >1 cm)	0.863	0.534–1.428	0.124	2.756	1.449–5.229	0.002
Ascites (absence vs. presence)	0.765	0.438–1.226	0.114	0.842	0.528–1.523	0.211

**Fig. 3:**
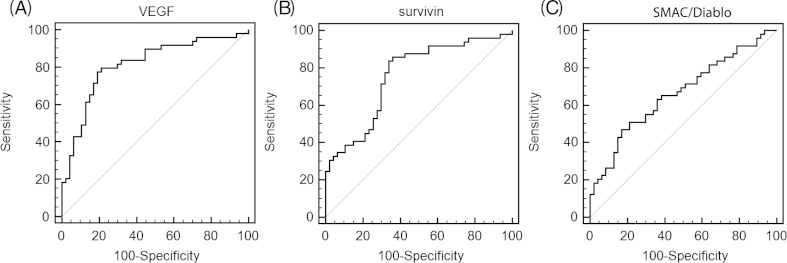
ROC curves of **a** VEGF, **b** survivin, and **c** Smac/DIABLO. ROC curves were derived by plotting the relationship between the specificity and the sensitivity at various cutoff levels. The area under the curve was 0.787 for VEGF, 0.698 for survivin, and 0.671 for Smac/DIABLO

Receiver operating characteristic (ROC) curve analyses unveiled that the serum level of VEGF was a useful biomarker to be used for distinguishing patients with serous ovarian carcinoma from controls within ROC curve areas of 0.787 (95 % CI = 0.659–0.839). At the cutoff value of 345 pg/ml for VEGF, the sensitivity was 83 %, and the specificity was 65 % (Fig. 3a). The ROC curve areas for survivin and Smac/DIABLO equalled 0.698 (95 % CI = 0.577–0.772) and 0.671 (95 % CI = 0.568–0.764), respectively. At the cutoff value of 110 pg/ml for survivin and 75 pg/ml for Smac/DIABLO, the sensitivity amounted to 82 and 62 %, while specificity amounted to 43 and 87 % (Fig. 3b, c).

## Discussion

Based on recent studies, it can be concluded that the inhibition of apoptosis, rather than enhanced cellular proliferation, is supposed to be a critical feature that might lead to ovarian carcinogenesis. Not to mention other regulators of ovarian cancer apoptosis, preoccupation has recently been addressed to survivin, a multifunctional member of the IAP gene family that both neutralizes cell death and regulates mitotic progression. Selective overexpression of survivin has been connected with higher tumor grade, advanced disease stage, rapid tumor progression, short patient survival, as well as resistance to therapy in patients with various malignancies [15, 16]. Some articles showed that survivin is a novel growth factor-inducible protective gene expressed by endothelial cells during angiogenesis and that survivin was initiated by VEGF via a PI3 K/Akt pathway [17].

Although platelet counts may have a great impact on serum VEGF levels, it has been shown that platelet-derived VEGF also reflects the biology of cancer cells and that serum appears to be more useful than plasma for the measurement of circulating VEGF in cancer patients for prognosis [18, 19]. That is why, in the present study, serum samples were used. Upon VEGF discovery till 2014, nine studies which directly linked preoperative serum levels of VEGF with ovarian cancer outcome were published. Unfortunately, due to the heterogeneity of these studies as well as lacking or incomplete information, there is no possibility to pool data and to perform a meta-analysis so as to receive indisputable indications referring to serum VEGF’s prognostic value. Even though seven [20–26] out of nine studies used the same VEGF assay, one [21] of them measured significantly lower VEGF values in ovarian cancer patients. Last but not the least, widely differing VEGF cutoff values (ranging from 100 to 826 pg/ml) were selected for univariate and multivariate analysis, depending on the statistical methods used for the analysis. Furthermore, seven out of eight studies, concerning the relation between VEGF and FIGO stage, emphasized that VEGF concentrations measured in sera were not connected with staging. In our study, the median level of VEGF in patients with serous ovarian cancer was significantly higher than that in normal volunteers, and also the median serum level of VEGF inversely correlated both with the progression of the stage and the increase of the grade of cancer. That may indicate that the effects promoted by VEGF are a continuous process and are independent of the clinical progression of the disease [27]. Previous studies relating serum VEGF levels to survival provided mixed results. Some studies found VEGF to be an independent prognostic factor by means of multivariate analysis [20, 22]. Other studies, on the other hand, did not find a correlation between serum VEGF and survival [25]. In our present study, preoperative serum VEGF levels were an independent prognostic factor for ovarian serous carcinoma. Therefore, in this examined uniform group, VEGF turns out to be the best prognostic marker for OS in comparison with the established prognostic variables such as stage and residual tumor size and appears to be an independent prognostic factor not only for DFS but also for tumor stage and grade.

Up till now the majority of the studies investigating the influence of survivin on the prognosis of ovarian cancers focused on tissue expression and discovered that survivin overexpression in tumors is associated with advanced disease stage, poorer survival, and chemotherapy or radiation resistance [28–31]. This is why it appears that the serum level of survivin might be advantageous in both the diagnosis and prognosis of cancer. Our results suggest that survivin is connected with progression and recurrence of serous ovarian cancer. In the present study, high serum levels of survivin were in close correlation with advanced stage, grade, presence of ascites, and level of cytoreduction. Previous studies revealed that serum survivin levels are associated with peritoneal metastasis of serous ovarian cancer [15], nodal involvement in breast cancer [32], advanced stages of head and neck cancer [33], and prostate cancer [34]. Moreover, survivin may suppress apoptosis by facilitating sequestration of the Smac/DIABLO, which antagonizes the inhibitory function of XIAP [35]. The role of this protein during carcinogenesis must still be investigated [36]. Nevertheless, Smac/DIABLO mRNA levels were found to be notably lower in lung cancers when compared to normal tissues. Patients with low Smac/DIABLO mRNA levels were shown to have worse prognosis [37].

Mizutani et al. [38] reported for the first time that Smac/DIABLO can be found in the serum and that the average serum level of this protein inversely correlated not only with the progression of the stage but also with the rise of the degree of bladder cancer. The present study has demonstrated for the first time that the serum level of Smac/DIABLO predicted the clinical outcome and that the lower levels were associated with poor prognosis in patients with serous ovarian cancer. Although Smac/DIABLO is not a secretory molecule, it is possible that it could be detected in serum. Its presence in the circulation may be due to the physiological cell death of normal tissues and cell death of tumor cells. In more aggressive tumors, the less apoptotic cell death occurs and less Smac/DIABLO is freed into the circulation. This finding is in accord with Mizutani et al. [38] hypothesis, but further studies are essential to determine the origin of this protein.

To conclude, the results of this study indicate that preoperative measurement of serum VEGF, survivin, and Smac/DIABLO may be of great help in early detection of serous ovarian cancer but also may provide important information about the patients’ outcome and prognosis. These precursory findings on the diagnostic and prognostic potential of VEGF, survivin, and Smac/DIABLO in serous ovarian carcinoma should now be confirmed in large prospective trials.

## Abbreviations

DFS: Disease-free survival
ELISA: Enzyme-linked immunosorbent assay
HR: Hazard ratio
IAP: Inhibitor of apoptosis
MDD: Minimum detectable dose
OS: Overall survival
ROC: Receiver operating characteristic
Smac/DIABLO: Second mitochondria-derived activator of caspase
TNF-α: Tumor necrosis factor alpha
VEGF: Vascular endothelial growth factor
